# Brain-inspired cognitive state modeling of complex English discourse reading: an LLM-parameterized hierarchical semi-Markov approach

**DOI:** 10.3389/fnbot.2026.1930227

**Published:** 2026-09-18

**Authors:** Bing Xie, Yongjin Hu, Linlin Zhao

**Affiliations:** 1 School of Foreign Studies, Suzhou University, Suzhou, China; 2 Suzhou No. 1 Junior High School, Suzhou, China

**Keywords:** brain-inspired cognitive modeling, cognitive state trajectory, complex discourse comprehension, English reading cognition, hierarchical hidden semi-Markov model, large language models

## Abstract

**Introduction:**

Complex English discourse reading requires readers to integrate lexical access, syntactic parsing, local meaning construction, cross-sentence inference, and discourse monitoring over time.

**Methods:**

We developed a brain-inspired, large-language-model-parameterized hierarchical hidden semi-Markov model that encodes lexical-, sentence-, and discourse-level representations and maps contextual embeddings to state-emission, transition, and duration parameters. Discourse-structure and cognitive-load constraints were incorporated into global decoding.

**Results:**

Experiments on 1,200 English reading passages achieved a macro-F1 of 0.860, a boundary accuracy of 0.83, a duration MAE of 0.52, and a discourse-consistency correlation of 0.76.

**Discussion:**

The results suggest that duration-aware latent-state modeling can provide an interpretable computational account of adaptive discourse comprehension while preserving explicit temporal structure.

## 1 Introduction

Complex English discourse comprehension is not reducible to word recognition or sentence-level interpretation. During continuous reading, readers build and revise layered mental representations, including surface encoding, propositional meaning, and situation models. Local cohesion, global coherence, and knowledge-based inference jointly shape this process (Graesser et al., 1994; Kintsch, 1988; van Dijk and Kintsch, 1983; Zwaan and Radvansky, 1998). For English reading research, a computational model that follows these evolving representations is valuable because it links cognitive theory with process diagnosis, reading assessment, and the analysis of discourse-level difficulty.

Several issues remain unresolved. First, comprehension unfolds as a temporal process. Event shifts in characters, time, space, causality, and goals can reorganize the reader's situation model, so the relevant cognitive state depends on earlier and later discourse context rather than on the current word alone. Second, many experimental studies still rely on short or isolated texts, which limits their ability to describe state persistence in coherent discourse. Third, eye movements and reading times provide online evidence of processing, but conventional analyses often emphasize local predictors and are less suited to estimating state boundaries and duration intervals (Demberg and Keller, 2008; Just and Carpenter, 1980; Levy, 2008; Rayner, 1998; Smith and Levy, 2013). Finally, lexical access, syntactic parsing, discourse representation, and eye-movement control have often been modeled separately, even though readers coordinate these processes during actual comprehension (Engbert et al., 2005; Kuperberg and Jaeger, 2016; Reichle et al., 1998).

Prior work offers useful but incomplete foundations. Cognitive theories of discourse comprehension clarify how coherence, inference, and prior knowledge guide meaning construction, yet they do not by themselves yield a unified latent-state decoding model. Probabilistic sequence models, including hidden Markov and hidden semi-Markov models, provide a principled way to infer unobserved states and state durations (Bishop, 2006; Rabiner, 1989; Yu, 2010), but their parameters are often fixed or only weakly tied to discourse semantics. Transformer-based language models provide strong contextual representations (Brown et al., 2020; Devlin et al., 2019; He et al., 2021; Vaswani et al., 2017), and recent model-brain studies indicate that large language models can serve as computational hypotheses about human language processing (Caucheteux and King, 2022; Goldstein et al., 2022; Pereira et al., 2018; Schrimpf et al., 2021; Wehbe et al., 2014; Yu et al., 2024). At the same time, brain-inspired accounts of efficient coding and predictive processing emphasize hierarchical, temporally constrained inference (Barlow, 1961; Clark, 2013; Friston, 2010; Rao and Ballard, 1999), which is closely aligned with the Research Topic's concern with adaptive large models.

This study therefore develops a large-language-model-parameterized hierarchical hidden semi-Markov model for complex English discourse reading. The model organizes discourse at lexical, sentence, and discourse levels; defines lexical activation, syntactic integration, semantic connection, cross-sentence inference, and discourse monitoring as duration-constrained latent cognitive states; and uses contextual embeddings from a large language model to generate state-emission, transition, and duration parameters. Discourse-structure consistency and cognitive-load constraints are then introduced during global decoding. The contribution is threefold. First, the model integrates local and discourse-level comprehension processes into a shared latent state space. Second, it connects large-model contextual representation with explicit cognitive-state duration, making the model more sensitive to the temporal persistence of comprehension. Third, it offers a brain-inspired framework in which large language models serve as parameter generators for interpretable cognitive trajectories. More broadly, the convergence of artificial intelligence, cognitive science, and neuroscience has motivated brain-inspired computational frameworks for formulating and testing hypotheses about human cognition (Hassabis et al., 2017; Lake et al., 2017; Kriegeskorte and Douglas, 2018; Richards et al., 2019).

## 2 Cognitive modeling of complex discourse reading

### 2.1 Overall system framework

The proposed framework treats complex discourse reading as a computational chain that connects hierarchical text input, contextual semantic encoding, state-parameter generation, and constrained decoding. The input layer organizes each passage into lexical, sentence, and paragraph units. The representation layer extracts contextual meaning and inter-sentence relations at each reading position. The inference layer then decodes the cognitive-state trajectory under discourse-structure and cognitive-load constraints. Formally, the multi-granular input of a passage is defined in Equation 1:


$$\begin{array}{l} \begin{array}{c} \text{D}=\left\{{\text{w}}_{1},{\text{w}}_{2},\ldots ,{\text{w}}_{\text{T}}\right\},\text{ S}=\left\{{\text{s}}_{1},{\text{s}}_{2},\ldots ,{\text{s}}_{\text{M}}\right\},\text{ P}=\left\{{\text{p}}_{1},{\text{p}}_{2},\ldots ,{\text{p}}_{\text{N}}\right\} \end{array} \end{array}$$ (1)

In formula (1), D represents the linear input sequence at the lexical level of the entire text, w_t_ represents the t-th lexical unit, and T represents the total number of lexical units; S represents the structural sequence at the sentence level, s_m_ represents the m-th sentence unit, and M represents the total number of sentences; *P* represents the sequence of text units at the paragraph level, p_n_ represents the n-th paragraph unit, and N represents the total number of paragraphs. This representation provides a unified input basis for subsequent modeling, ensuring that each reading position is within a clear lexical, sentence, and text structure.

The central component of the system is the contextual encoder. Rather than assigning fixed probabilities to cognitive states, the model uses a large language model to encode local lexical cues, inter-sentence cohesion, and discourse organization around the current reading position. These representations are mapped to the conditional parameters required by the semi-Markov module. State emission, transition, and duration distributions are therefore adjusted by the current discourse environment, which helps keep cognitive-state inference aligned with the text being read.

In this architecture, hierarchical text input provides the observable reading sequence, large-model encoding supplies context-sensitive semantic evidence, the hierarchical hidden semi-Markov module represents state persistence and switching, and structural constraints produce a globally coherent cognitive trajectory. The information flow among these components is shown in Figure 1.

**Figure 1:**
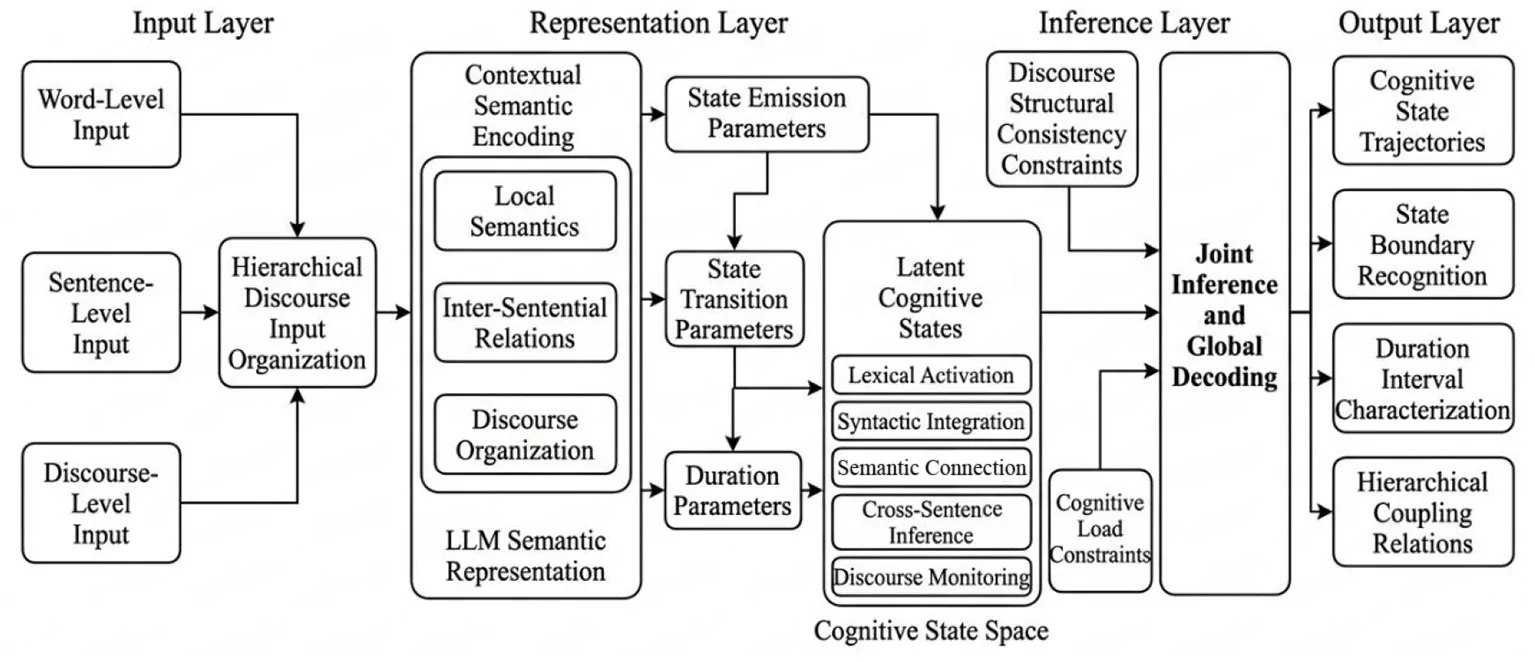
Cognitive modeling framework for complex discourse reading.

### 2.2 Hierarchical discourse representation and cognitive state space

This section focuses on the model's input organization and state representation, with the aim of defining an explicit mapping among reading position, discourse level, and cognitive-processing state. The lexical sequence D, sentence sequence S, and paragraph sequence P, using reading position t as a unified index, compress the information from the three levels into the same representation space, forming a hierarchical semantic vector, as shown in formula (2):


$$\begin{array}{l} {\text{x}}_{\text{t}}=[e_{t}^{\left(w\right)}//e_{t}^{\left(s\right)}//e_{t}^{\left(p\right)}] \end{array}$$ (2)

In formula (2), *x*_t_ represents the comprehensive representation of the t-th reading position; ${\text{e}}_{\text{t}}^{\left(\text{w}\right)}$ represents the lexical layer representation of that position, used to carry local word meaning and adjacent semantic clues; ${\text{e}}_{\text{t}}^{\left(\text{s}\right)}$ represents the sentence layer representation, used to carry syntactic structure and intra-sentence propositional relationships; ${\text{e}}_{\text{t}}^{\left(\text{p}\right)}$ represents the discourse layer representation, used to carry cross-sentence connection, paragraph progression and overall coherence information; the symbol // represents the vector concatenation operation. Through this representation, any reading position simultaneously retains both local content and the higher-level discourse background, so that subsequent state determination no longer depends on single-layer features.

The main processing stages in complex discourse reading are represented as a finite set of latent cognitive states. The state set is shown in Equation 3:


$$\begin{array}{l} \begin{array}{c} Z=\left\{{\text{z}}_{1},{\text{z}}_{2},{\text{z}}_{3},{\text{z}}_{4},{\text{z}}_{5}\right\}=\left\{\text{Lex},\text{Syn},\text{Sem},\text{Inf},\text{Mon}\right\} \end{array} \end{array}$$ (3)

In formula (3), the cognitive state space comprises five states: lexical activation for word recognition, syntactic integration for intra-sentence structural analysis, semantic connection for local meaning integration, cross-sentence inference for inter-sentence linkage, and discourse monitoring for coherence checking. This definition transforms the reading process from an abstract psychological activity into a sequence of latent states whose boundaries are inferred by the downstream model rather than assumed by the state definition itself.

The relationship between hierarchical representation and cognitive state is not a simple correspondence, but a joint mapping of “multi-layer semantic input—latent state determination.” For any reading position t, the model uses *x*_t_ lexical layer information to identify the current local activation intensity, sentence layer information to determine the intra-sentence integration needs, and discourse layer information to determine cross-sentence reasoning and monitoring pressure. Then, it determines a more suitable processing state within the state space defined by formula (3). The state space constructed in this way not only retains the hierarchical features of complex discourse comprehension, but also provides a clear observation interface and state boundary for subsequent large language model parameterization and hidden semi-Markov modeling. The differences in hierarchical response at different reading positions and their mapping relationship to the cognitive state space are shown in Figure 2.

**Figure 2:**
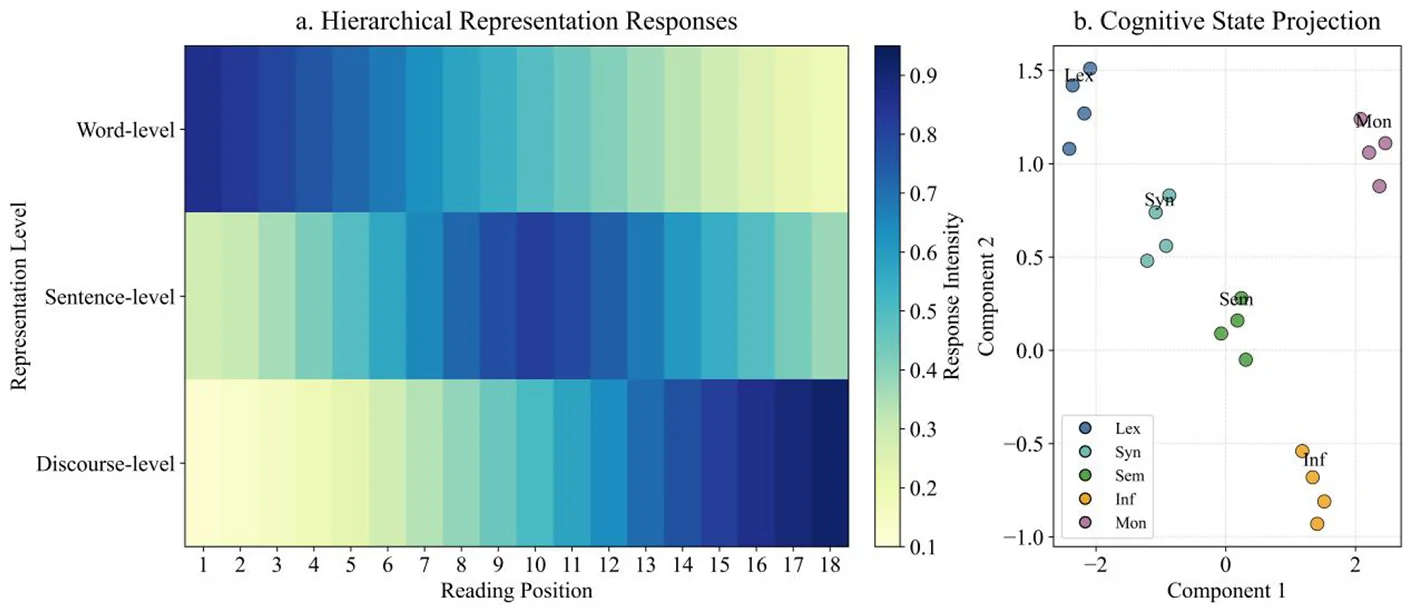
Distribution of discourse hierarchical representation and cognitive state mapping features. **(a)** hierarchical representation response intensity. **(b)** cognitive state projection distribution.

Figure 2 presents the representational basis of the model. The left panel shows that the dominant source of information shifts gradually from lexical cues to sentence- and discourse-level representations as reading proceeds. The right panel shows that the five cognitive states form distinguishable regions in the projected representation space. This pattern suggests that joint encoding of lexical information, intra-sentence structure, and discourse organization retains useful cues for state discrimination, while avoiding a one-to-one reduction of states to surface linguistic units. In Figure 2a, response intensity is the normalized contribution of each hierarchical layer at each reading position, computed by applying the normalization function over the squared norms of the lexical, sentence, and discourse components of the joint representation and averaging the resulting shares within twenty equal discourse-position bins. Reading positions are aligned across texts by rescaling each passage to a common axis of one hundred normalized positions, and the panel reports averages across all 180 test passages rather than a single example or a simulated pattern. Figure 2b is produced with uniform manifold approximation and projection applied to the 768-dimensional contextual representations of the 77,220 test positions, and each plotted point denotes one reading position colored by its decoded state. The projection is fitted without access to the state labels, so supervision from the labels plays no role in the geometry of the panel. As quantitative evidence of separability, the projected clusters reach a silhouette score of 0.41, and a logistic-regression probe trained on half of the projected positions and evaluated on the held-out half reaches a five-way accuracy of 0.79, with both statistics computed on the test split only.

### 2.3 LLM-parameterized hierarchical hidden semi-Markov modeling

A large language model is introduced as a parameter generator within the hierarchical hidden semi-Markov framework. In this design, latent cognitive states are not governed by a static probability table. Instead, the contextual representation at each reading position determines the emission strength, transition bias, and expected state duration. This choice reflects the assumption that reading cognition is adaptive: the same lexical or syntactic cue may trigger different processing demands when it appears in different discourse contexts. Given the whole reading sequence, the contextual representation of position t is obtained as shown in Equation 4:


$$\begin{array}{l} \begin{array}{c} {\text{h}}_{\text{t}}=\text{LLM}\left({\text{x}}_{1},{\text{x}}_{2},\ldots ,{\text{x}}_{\text{t}},\ldots ,{\text{x}}_{\text{T}}\right) \end{array} \end{array}$$ (4)

In formula (4), *h*_t_ represents the contextual semantic representation of the t-th reading position, *LLM*(·) represents the large language model encoding function, *x*_1_, *x*_2_, …, *x*_t_, …, *x*_T_ represents the hierarchical input sequence of the entire text on the reading time axis, and T represents the total number of reading positions. The left side of formula (4) gives the semantic encoding result of position t, and the right side explains that the result is jointly determined by the entire sequence context. The result obtained thus *h*_*t*_ simultaneously preserves local lexical clues, inter-sentence dependencies, and textual organization information, providing a unified condition for subsequent parameter generation.

In state-emission modeling, the probability of the observed variable *y*_*t*_ given cognitive state *z*_*t*_ = *k* is written in a context-driven parameterized form, as shown in Equation 5:


$$\begin{array}{l} \begin{array}{c} \text{p}\left({\text{y}}_{\text{t}}\;|\;{\text{z}}_{\text{t}}=\text{k}\right)=\text{Softmax}\left({\text{W}}_{\text{e}}^{\left(\text{k}\right)}{\text{h}}_{\text{t}}+{\text{b}}_{\text{e}}^{\left(\text{k}\right)}\right) \end{array} \end{array}$$ (5)

In formula (5), *p*(*y*_t_ | *z*_t_ = *k*) represents the conditional emission probability of position t in state k, *y*_t_ represents the observed response at that position, *z*_t_ represents the latent cognitive state of position t, ${\text{W}}_{\text{e}}^{\left(\text{k}\right)}$ and $b_{e}^{\left(k\right)}$ represent the emission mapping matrix and bias vector corresponding to state k, respectively, and *Softmax*(·) represents the probability normalization function. This indicates that the explanatory strength of the state for the observation is no longer a fixed setting, but is adjusted according to the semantic environment of the current position. In the dataset, the observed response at position t is a five-dimensional vector whose k-th entry is the proportion of annotation decisions at that position assigned to state k, so each entry lies between zero and one and the five entries sum to one. The emission mapping matrix of state k maps the 256-dimensional state representation to five output logits, the bias vector has five entries, and the normalization function in Equation 5 is applied across the five state categories. The manually annotated labels serve as the supervision targets for the latent state sequence during training, while the observed response vector supplies the empirical profile against which the emission distribution is fitted, so the latent state, the observation, and the supervision target remain three distinct quantities coupled through the joint objective in Equation 8.

For state-transition modeling, the contextual representation is further used to estimate dynamic transition biases between adjacent states, as shown in Equation 6:


$$\begin{array}{l} \begin{array}{c} {\text{a}}_{\text{ij}}^{\left(\text{t}\right)}=\text{p}\left({\text{z}}_{\text{t}}=\text{j }|\;{\text{z}}_{\text{t}-1}=\text{i}\right)=\frac{\exp\left({\text{u}}_{\text{ij}}^{⊤}{\text{h}}_{\text{t}}+{\text{b}}_{\text{ij}}\right)}{\sum_{{\text{j}}^{\text{′}}}\exp\left({\text{u}}_{\text{i}{\text{j}}^{\text{′}}}^{⊤}{\text{h}}_{\text{t}}+{\text{b}}_{\text{i}{\text{j}}^{\text{′}}}\right)} \end{array} \end{array}$$ (6)

In formula (6), ${\text{a}}_{\text{ij}}^{\left(\text{t}\right)}$ represents the conditional probability of position t transitioning from state i to state j, *z*_t−1_ and *z*_t_ represents the implicit cognitive states of two adjacent reading positions, respectively. ${\text{u}}_{\text{ij}}$ represents the state transition weight vector, *b*_ij_ represents the corresponding bias term, and *j*′ represents all candidate target states. Thus, the cognitive state switching process is written as a dynamic evolutionary process constrained by the discourse environment.

Because cognitive processing in reading often persists over several units rather than changing at every position, state duration is explicitly modeled in addition to transition probability, as shown in Equation 7:


$$\begin{array}{l} \begin{array}{c} \text{p}\left({\text{d}}_{\text{t}}=\text{l }|\;{\text{z}}_{\text{t}}=\text{k}\right)=\frac{\exp\left({\text{v}}_{\text{k}}^{⊤}{\text{h}}_{\text{t}}+{\text{γ}}_{\text{k},\text{l}}\right)}{\sum_{{\text{l}}^{\text{′}}=1}^{{\text{L}}_{\max}}\exp\left({\text{v}}_{\text{k}}^{⊤}{\text{h}}_{\text{t}}+\gamma_{\text{k},{\text{l}}^{\text{′}}}\right)} \end{array} \end{array}$$ (7)

In formula (7), *p*(*d*_t_ = *l* | *z*_t_ = *k*) represents the conditional probability that position t is in state k with a duration of length l, *d*_t_ represents the state duration span, ${\text{v}}_{\text{k}}$ represents the duration weight vector of state k, *γ*_*k,l*_ represents the bias term of state k at length l, and *L*_max_ represents the maximum duration. This modeling allows the model to not only identify the state to which the current position belongs, but also to characterize the state maintenance interval. The five state categories and their annotation guidelines are defined *a priori* from discourse-comprehension theory, whereas all emission weights, transition weight vectors, duration weight vectors, and bias terms are estimated from the annotated training set by optimizing the joint objective. No transition path, duration length, or state ordering is hard-coded into the parameterization. The transition matrix is initialized uniformly, and the duration parameters start from a shared flat prior, so any directional structure in the decoded trajectories reflects parameter values learned from the data rather than fixed architectural assumptions. To verify this property, we ran a sensitivity analysis in which the duration term, the discourse-structure consistency constraint, and the ordering-based initialization were removed or randomized one at a time, and the decoded trajectories were re-estimated on the test set.

All three types of state parameters are driven by *h*_*t*_ through a unified mechanism. Therefore, the implicit cognitive states in the model retain the ability of hierarchical hidden semi-Markov structures to express temporal processes, while also absorbing the modeling advantages of large language models for complex discourse semantic dependencies. The emission response and duration variation characteristics of different cognitive states in the reading sequence are shown in Figure 3.

**Figure 3:**
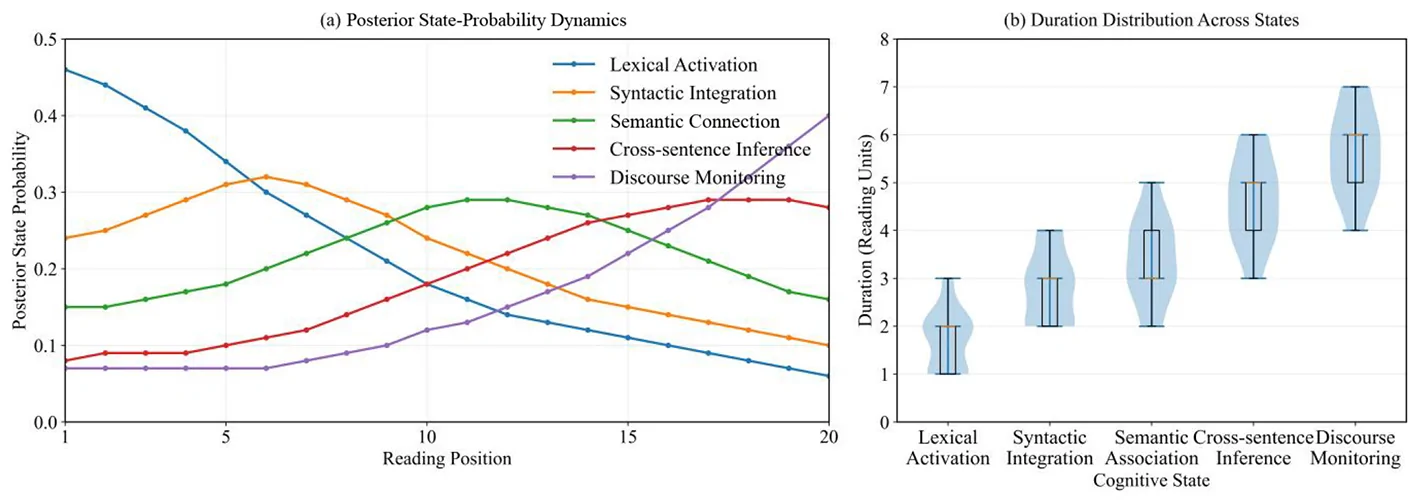
Cognitive-state posterior probabilities and decoded duration distributions. **(a)** posterior state-probability dynamics of the five cognitive states on the test set. **(b)** decoded duration distributions of the five cognitive states on the test set.

Figure 3 shows that cognitive states differ not only in category but also in temporal organization. The left panel indicates that the dominant response gradually moves from lower-level processing to higher-level integration. The right panel shows that higher-level comprehension states tend to last longer, whereas lexical activation is more often short and local. These results support the use of a duration-aware latent-state model: it captures both switching and persistence, two features that are difficult to represent with ordinary position-wise classifiers. Each curve in Figure 3a is the posterior probability of the corresponding cognitive state given the contextual representation, obtained by applying the normalization function across the five states at each position, and the five curves therefore sum to one at every reading position. The curves do not depict the conditional emission probability of the observation under each state, and the figure caption and axis label have been revised accordingly. Both panels of Figure 3 report model outputs on the test set rather than empirical annotation statistics or illustrative simulations. Figure 3a averages the posterior probabilities over the 180 test passages within twenty equal discourse-position bins and applies a moving average over three adjacent bins for smoothing. Each violin in Figure 3b summarizes the durations of the decoded segments assigned to the corresponding state across all test passages, so the violins depict decoded durations from the fitted model rather than annotated durations or the duration distributions of Equation 7.

### 2.4 Constrained joint inference and cognitive-trajectory generation

The decoding stage combines observation fitting, state evolution, and duration modeling in a single objective. This joint inference mechanism follows the logic of probabilistic sequence modeling while adding discourse-level constraints that are specific to reading comprehension (Bishop, 2006; Rabiner, 1989; Yu, 2010). The objective is defined in Equation 8:


$$\begin{array}{l} \begin{array}{c} \text{J}=\overset{\text{T}}{\underset{\text{t}=1}{\sum }}\log\text{p}\left({\text{y}}_{\text{t}}\;|\;{\text{z}}_{\text{t}}\right)+{\text{λ}}_{1}\overset{\text{T}}{\underset{\text{t}=2}{\sum }}\log{\text{a}}_{{\text{z}}_{\text{t}-1}{\text{z}}_{\text{t}}}^{\left(\text{t}\right)}+{\text{λ}}_{2}\overset{\text{T}}{\underset{\text{t}=1}{\sum }}\log\text{p}\left({\text{d}}_{\text{t}}\;|\;{\text{z}}_{\text{t}}\right) \end{array} \end{array}$$ (8)

In formula (8), *J* represents the global objective function, T represents the total number of reading positions, *y*_*t*_ represents the observed response at the t-th position, *z*_*t*_ represents the corresponding implicit cognitive state, ${\text{a}}_{{\text{z}}_{\text{t}-1}{\text{z}}_{\text{t}}}^{\left(\text{t}\right)}$ represents the state transition probability between adjacent positions, *d*_*t*_ represents the state duration, λ_1_ and λ_2_ represent the weight coefficients of the transition term and the duration term, respectively.

To reduce locally plausible but globally incoherent paths, two constraints are added: discourse-structure consistency and cognitive-load control. These constraints are shown in Equation 9:


$$\begin{array}{l} \begin{array}{c} {ℒ}_{\text{c}}=\alpha \;{ℒ}_{\text{disc}}+\beta \;{ℒ}_{\text{load}} \end{array} \end{array}$$ (9)

In formula (9), ℒ_*c*_ represents the overall constraint term, ℒ_*disc*_ represents the discourse structure consistency constraint, used to characterize the coordination relationship between the state boundary and the sentence structure, ℒ_*load*_ represents the cognitive load constraint, used to limit the non-stationary fluctuations of state intensity and switching frequency, α and β are the corresponding weight parameters. The optimal cognitive state sequence is obtained through global decoding, as shown in (Equation 10):


$$\begin{array}{l} \begin{array}{c} \hat{\text{Z}}=\text{arg}\underset{\text{Z}}{\max}\left(\text{J}-{ℒ}_{\text{c}}\right) \end{array} \end{array}$$ (10)

In (Equation 10), *Z* denotes the set of candidate state paths, whereas $\hat{Z}$ denotes the optimal cognitive trajectory; argmax selects the state sequence that maximizes the objective over all candidate paths. This indicates that the optimal trajectory is jointly determined by the joint objective and global constraints.

The model finally outputs a cognitive-state trajectory, state boundaries, and duration intervals for the whole passage. Parameterized state modeling and global constraints are thereby brought into one framework, allowing complex discourse reading to be represented as a temporally continuous and cognitively interpretable process. The decoded trajectory and hierarchical coupling pattern are shown in Figure 4.

**Figure 4:**
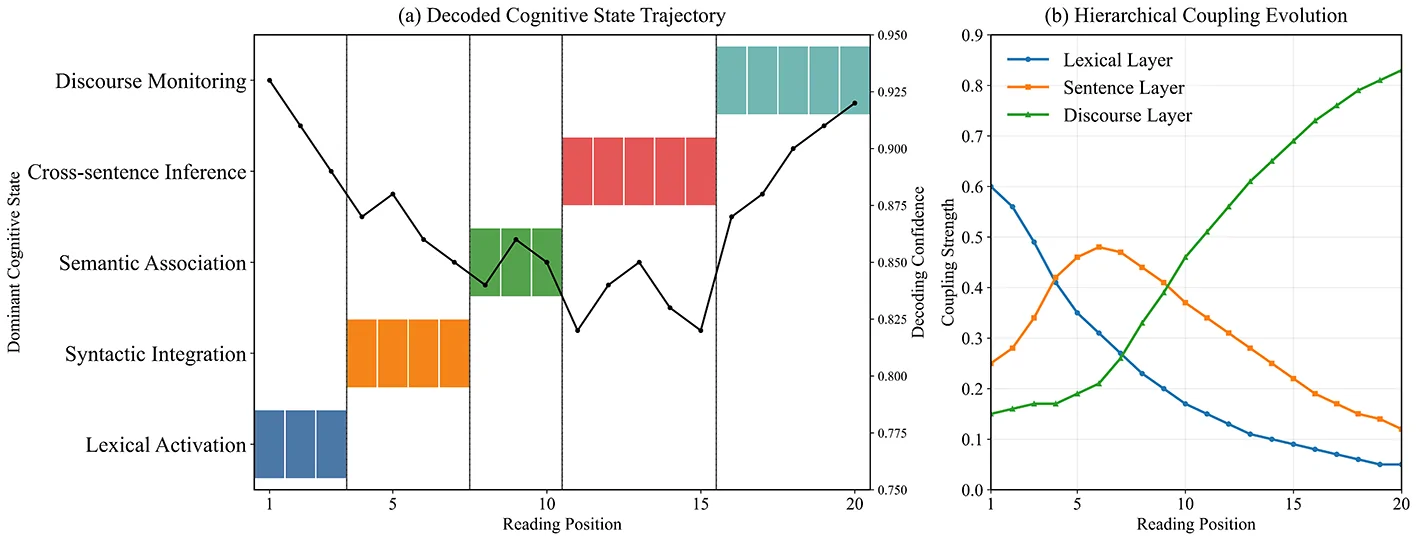
Cognitive-state trajectory and hierarchical coupling evolution. **(a)** Cognitive-state trajectory after decoding. **(b)** Hierarchical coupling evolution.

Figure 4 links the decoded state sequence with the changing contribution of lexical, sentence, and discourse representations. The left panel shows that dominant states move from local processing toward high-level integration, while the right panel indicates that discourse-level support becomes stronger as comprehension progresses. This pattern is consistent with the view that readers begin with lexical access and intra-sentence organization but increasingly rely on accumulated inter-sentence relations and global coherence checks. The figure also illustrates why structural constraints are necessary: without them, a model may fit local cues while producing state jumps that are unlikely in continuous reading. In Figure 4a, the colored blocks represent the decoded dominant cognitive state at each reading position, and the black line represents decoding confidence, defined as the maximum posterior state probability at that position. The displayed trajectory is aggregated across all test passages by rescaling each decoded sequence to twenty normalized reading positions and assigning to each position the modal dominant state across passages. The vertical arrangement of the five states follows the processing order proposed in construction-integration theory and later discourse-comprehension models, under which surface and lexical processing precede propositional integration and situation-model updating. This ordering enters the model only through the initialization of adjacent-state transition candidates and the layout of the figure. It is not enforced during decoding, because every ordered state pair retains a learned nonzero transition parameter, and the sensitivity analysis in **Section 4.2** shows that the forward progression survives when the ordering-based initialization is removed. The five states remain categorical, and the trajectory is interpreted as a summary of decoded label sequences rather than as evidence of a continuous hierarchical dimension.

## 3 Experimental design

### 3.1 Corpus sources and task definition

The experimental corpus was constructed to support cognitive-process modeling of English reading. It includes TOEFL iBT academic reading passages, IELTS academic reading passages, Cambridge CAE/CPE reading passages, and continuous English expository and narrative passages. After screening, 420, 360, 240, and 180 texts from these four sources were retained, yielding 1,200 valid passages. From 1,386 candidate texts, 186 were removed because of incomplete structure, residual question stems, duplicate content, or paragraph-boundary problems. All retained texts preserve complete discourse structures, and lexical, sentence, and paragraph boundaries were checked to ensure stable hierarchical alignment.

Discourse complexity was defined along two dimensions: linguistic complexity and textual complexity. Linguistic complexity was measured through sentence length, syntactic embedding, lexical difficulty, and dependency density. Textual complexity was measured through referential-chain span, inter-sentence cohesion, logical-relation density, and thematic-progression distance. This operationalization follows established work on discourse coherence, inference, readability, and cohesion analysis (Graesser et al., 2004; McNamara and Kintsch, 1996), while also reflecting the predictive and expectation-based nature of online language comprehension (Kuperberg and Jaeger, 2016; Levy, 2008). Each of the eight complexity measures is computed per passage and converted to a z-score with the mean and standard deviation of the full corpus. The linguistic-complexity score of a passage is the unweighted mean of its four linguistic z-scores, and the textual-complexity score is the unweighted mean of its four textual z-scores. Passages are assigned to the low, medium, and high groups by tertiles of the corresponding score computed within each source collection, which keeps the three groups comparable across sources, and the resulting group sizes are reported in Table 1.

**Table 1:** Experimental corpus statistics, complexity distribution, and task configuration.

Section	Item	Train	Validation	Test	Overall/configuration
Corpus statistics	Number of passages	840	180	180	1,200
	Lexical units per passage	428.0 ± 67.4	431.2 ± 69.1	429.0 ± 68.3	429.1 ± 67.9
	Sentential units per passage	19.4 ± 3.8	19.7 ± 3.9	19.4 ± 3.7	19.4 ± 3.8
	Paragraph units per passage	4.8 ± 1.1	4.9 ± 1.2	4.8 ± 1.1	4.8 ± 1.1
Hierarchical unit statistics	Total lexical units	359,520	77,580	77,220	514,320
	Total sentential units	16,296	3,546	3,492	23,334
	Total paragraph units	4,032	882	864	5,778
Linguistic complexity distribution	Low-complexity passages	238	52	50	340
	Medium-complexity passages	356	76	78	510
	High-complexity passages	246	52	52	350
Discourse complexity distribution	Low-complexity passages	224	49	47	320
	Medium-complexity passages	371	79	81	531
	High-complexity passages	245	52	52	349
Task configuration	Core tasks	—	—	—	State recognition; boundary detection; duration fitting; discourse consistency evaluation
State label distribution	Lexical activation labels	72,240	15,588	15,492	103,320
	Syntactic integration labels	90,864	19,512	19,404	129,780
	Semantic connection labels	82,368	17,784	17,628	117,780
	Cross-sentence inference labels	65,352	14,112	14,040	93,504
	Discourse monitoring labels	48,696	10,584	10,656	69,936

Length statistics are reported as mean ± standard deviation.

The data were divided at the text level, so that no passage appeared in more than one split. The final training, validation, and test sets contained 840, 180, and 180 texts, respectively. The split achieved a text-level separation rate of 100.0%, a topic-stratification retention rate of 96.8%, and a post-split length-distribution divergence of 0.027. Although the corpus covers several standardized reading sources and complexity levels, it remains centered on controlled academic reading materials. Its generalizability to classroom reading, open online discourse, and multimodal reading environments therefore requires further validation.

Four tasks were defined on the basis of this corpus. Cognitive-state recognition identifies the dominant latent state at each reading position. State-boundary detection locates the start, end, and switching points of cognitive states. Duration fitting estimates how long each state persists. Discourse-comprehension consistency evaluates the match between the decoded cognitive trajectory and the passage's organizational structure. All four tasks operate on the manual annotations described in **Section 3.2**, and every label count reported in this paper refers to the manual annotations rather than to decoded model outputs. The operational decision criteria used to assign the five state labels are stated in **Section 3.2**, and the counts are broken down by data split in Table 1. Table 1 reports the corpus statistics, complexity distribution, and task configuration.

### 3.2 Cognitive state annotation and data preprocessing

A cognitive-state annotation scheme was developed for complex discourse reading. The reading position served as the basic unit, and each position received one dominant state label aligned across lexical, sentence, and discourse levels. The five state categories correspond to the model definition: lexical activation, syntactic integration, semantic connection, cross-sentence inference, and discourse monitoring. The annotation criteria focused on the processing function of the position within the comprehension sequence rather than on surface linguistic form alone.

A reading position is one lexical unit together with its aligned sentence and paragraph units. Lexical activation is assigned when the dominant demand at the position is word recognition or initial meaning access, operationalized through low lexical frequency or ambiguity with routine syntactic and discourse roles. Syntactic integration is assigned when the dominant demand is building or revising the phrase structure of the current sentence, operationalized through embedding depth, non-canonical order, or structural ambiguity at the position. Semantic connection is assigned when the dominant demand is integrating the position into the local propositional meaning of the current sentence. Cross-sentence inference is assigned when comprehension of the position requires information stated in earlier sentences or the recovery of an implicit relation not encoded within the current sentence. Discourse monitoring is assigned when the dominant function is checking global coherence, updating the situation model, or regulating the reading process at paragraph or passage boundaries. When more than one function plausibly applies to a position, annotators select the state whose processing demand sits highest in the hierarchy, with ties resolved toward the function supported by explicit textual cues. During arbitration the third annotator applies the same criteria to each disputed position, and a final label is accepted only when it matches the choice of the third annotator and at least one of the two original annotators. Cohen's kappa is computed on the five-category labels over all annotated positions, and the category-specific values are one-versus-rest agreements for each state.

The annotation rules emphasized hierarchical consistency, sequence continuity, and boundary plausibility. Hierarchical consistency required annotators to consider local lexical information, intra-sentence structure, and discourse context together. Sequence continuity required neighboring labels to follow the normal progression of reading unless a clear semantic or structural shift occurred. Boundary plausibility required state onsets and offsets to align with sentence structure and discourse transitions. Three researchers trained in English reading research and computational linguistics completed the annotation. Trial annotation covered 120 texts and 51,480 reading positions; disagreements were used to refine the guidelines. In the formal stage, two annotators worked independently, cross-checked each text, and submitted unresolved cases to a third annotator. Trial-stage Cohen's kappa was 0.842. In the formal review sample of 180 texts and 77,220 positions, the overall agreement rate was 94.7%, the position-level disagreement rate was 7.6%, and the arbitration rate was 6.4%. Kappa values for lexical activation, syntactic integration, semantic connection, cross-sentence inference, and discourse monitoring were 0.881, 0.857, 0.841, 0.819, and 0.806, respectively.

Data preprocessing proceeded through text cleaning, hierarchical segmentation, reading-position alignment, and context construction. Text cleaning removed 4,127 noisy symbols. Sentence boundaries were identified with spaCy 3.7.2 and the en_core_web_trf 3.7.2 model. Position alignment linked word-level units with sentence and paragraph units, and manual correction was applied to word-boundary and paragraph-boundary mismatches, with correction rates of 3.8% and 2.6%. Context windows included the two preceding sentences, the current sentence, and the two following sentences, with a maximum input length of 512 tokens and a window step of 64. The paragraph-alignment success rate was 99.3%. After preprocessing, each sample followed the structure reading position, hierarchical representation, and state label. The formal review sample of 180 texts and 77,220 positions is exactly the test split reported in Table 1, and every lexical unit in the test set received one cognitive-state label, which is why the reported category counts sum to 77,220. All agreement and arbitration statistics are computed on this test split. The train, validation, and test split was fixed before guideline refinement began, and annotation, arbitration, and label finalization on the test set proceeded independently of model development, hyperparameter tuning, and error analysis, with no test label modified after model evaluation started.

### 3.3 Baseline models and training settings

To identify the source of performance gain, the comparison models were arranged in a progressive sequence. The baseline classifier tests how far local representation alone can support position-wise state discrimination. The sequence-labeling model adds adjacent-label dependence. The hidden-state model without duration modeling tests latent-state inference after removing explicit duration constraints. The proposed model adds large-language-model parameterization and semi-Markov duration modeling. This design separates gains from representation quality, sequence dependence, latent-state inference, and duration modeling, which is consistent with standard probabilistic modeling practice (Bishop, 2006; Rabiner, 1989; Yu, 2010) and with recent large-model approaches to contextual encoding (Devlin et al., 2019; He et al., 2021; Vaswani et al., 2017).

In terms of training implementation, all models used the “reading position—hierarchical representation—state label” structure preprocessed in **Section 3.2** as a unified input and shared the same data partitioning and evaluation protocol to control biases caused by non-model factors. The large language model's encoding part used DeBERTa-v3-base as the context representation, with an output dimension of 768. The hidden state space of the hierarchical hidden semi-Markov module was consistent with the five cognitive states defined in **Section 2.2**, with a state representation dimension of 256. Transition parameters and duration parameters were jointly learned within this space. During training, the AdamW optimizer (Loshchilov and Hutter, 2019) was used, with an initial learning rate of 2 × 10^−5^, a weight decay coefficient of 0.01, a batch size of 16, and a maximum training epoch of 30. The first 10% of training steps were used for linear warm-up, followed by cosine annealing to update the learning rate. To ensure experimental reproducibility, all models were trained using a fixed random seed, the same data partitioning, the same input window length, and a consistent early stopping criterion. To suppress parameter oscillations, the gradient clipping threshold was set to 1.0, the dropout ratio was set to 0.1, and early stopping was triggered when validation macro-F1 did not improve for four consecutive evaluation periods. For semi-Markov duration modeling, the maximum duration L was set to 12 reading units to ensure that the state interval modeling covered the main cognitive duration span while avoiding excessively long periods from interfering with decoding stability.

All baseline models were trained under the same hardware environment, random seed, data split, batch construction method, and input-window length. The platform used an NVIDIA RTX 4090 24GB GPU, an Intel Core i9-13900K CPU, 64GB RAM, CUDA 12.1, PyTorch 2.1.2, and Transformers 4.38.2. The random seed was 42. Each model was trained 3 times independently. The proposed model required an average of 3.8 h per run; its standard deviations across runs were 0.004 for macro-F1, 0.006 for boundary accuracy, and 0.012 for duration MAE. These controls make the comparison depend primarily on model structure rather than incidental training conditions.

### 3.4 Evaluation metrics

The evaluation system was designed around the four tasks and therefore covers category discrimination, boundary localization, duration fitting, and discourse-level consistency. Two principles guided metric selection. First, each metric had to correspond directly to a task objective. Second, the metrics together had to reflect the dynamic nature of reading cognition, so that the evaluation would not be reduced to single-position classification accuracy.

For cognitive-state recognition, macro-F1 was used as the main metric. Precision and recall were calculated for each state and then averaged across states. This metric is appropriate because the five cognitive states are not equally frequent; macro-F1 prevents high-frequency categories from masking weaker performance on less frequent but cognitively important states.

For state-boundary detection, boundary accuracy measured whether the predicted start, end, and transition points matched the annotated boundaries within a unified tolerance range. Because state boundaries connect local recognition with the overall cognitive trajectory, higher boundary accuracy indicates a more stable segmentation of the reading process.

For duration modeling, mean absolute error (MAE) measured the difference between predicted and annotated state duration. A lower MAE indicates a closer fit to the persistence interval of the cognitive state. This metric is especially important for evaluating the semi-Markov component, since ordinary sequence labeling can identify state categories without explaining how long those states are maintained.

For discourse-comprehension consistency, Pearson's correlation coefficient measured the alignment between the decoded cognitive trajectory and discourse organization. The metric captures whether changes in state intensity and switching density correspond to the progression of the text. It therefore evaluates the explanatory value of the trajectory at the whole-sequence level.

## 4 Results

### 4.1 Cognitive state recognition

Using the same data split, input structure, and evaluation protocol, the proposed model was compared with the baseline classifier, sequence-labeling model, and hidden-state model without duration modeling. Performance was evaluated for lexical activation, syntactic integration, semantic connection, cross-sentence inference, and discourse monitoring. Macro-F1 was used to summarize multi-state recognition. Table 2 reports the results.

**Table 2:** F1 Comparison of different models on five cognitive state recognition tasks.

Model	Lex.	Syn.	Sem.	Cross.	Disc.	F1	LCL	UCL	p (boot)
Baseline classifier	0.782	0.761	0.744	0.713	0.701	0.74	0.733	0.747	< 0.001
Sequence labeling model	0.831	0.809	0.794	0.766	0.755	0.791	0.785	0.797	< 0.001
Hidden-state model without duration modeling	0.858	0.841	0.826	0.799	0.786	0.822	0.816	0.828	0.008
Proposed LLM-parameterized HHSMM	0.901	0.882	0.861	0.832	0.824	0.86	0.854	0.866	—

The numbers of test labels for lexical activation, syntactic integration, semantic connection, cross-sentence inference, and discourse monitoring are 15,492, 19,404, 17,628, 14,040, and 10,656, respectively. Lex., lexical activation; Syn., syntactic integration; Sem., semantic connection; Cross., cross-sentence inference; Disc., discourse monitoring; F1, Macro-F1 (unweighted average of the five state-wise F1 scores); LCL/UCL, lower/upper bounds of the 95% bootstrap confidence interval; p (boot), paired bootstrap *p*-value for difference relative to the proposed model; “—” denotes the reference model.

Table 2 shows that the proposed model reaches a macro-F1 of 0.860, outperforming the hidden-state model without duration modeling (0.822), the sequence-labeling model (0.791), and the baseline classifier (0.740). Lexical activation and syntactic integration obtain relatively high F1 scores of 0.901 and 0.882, suggesting that local lexical cues and intra-sentence structures are comparatively stable. Semantic connection reaches 0.861, indicating that local meaning integration benefits from contextual encoding. Cross-sentence inference and discourse monitoring score 0.832 and 0.824; these lower values are expected because higher-level states involve longer dependencies, sparser labels, and more ambiguous boundaries. Compared with the no-duration hidden-state model, the proposed model improves cross-sentence inference by 0.033 and discourse monitoring by 0.038, which indicates that duration modeling and structural constraints are especially helpful for high-level comprehension states.

### 4.2 State boundary detection and phase switching

This section evaluates whether the model can locate state transitions and preserve the directional organization of reading stages. All four models were tested with the same boundary labels and comparison settings. Figure 5 reports boundary deviations and the dominant state-switching paths.

**Figure 5:**
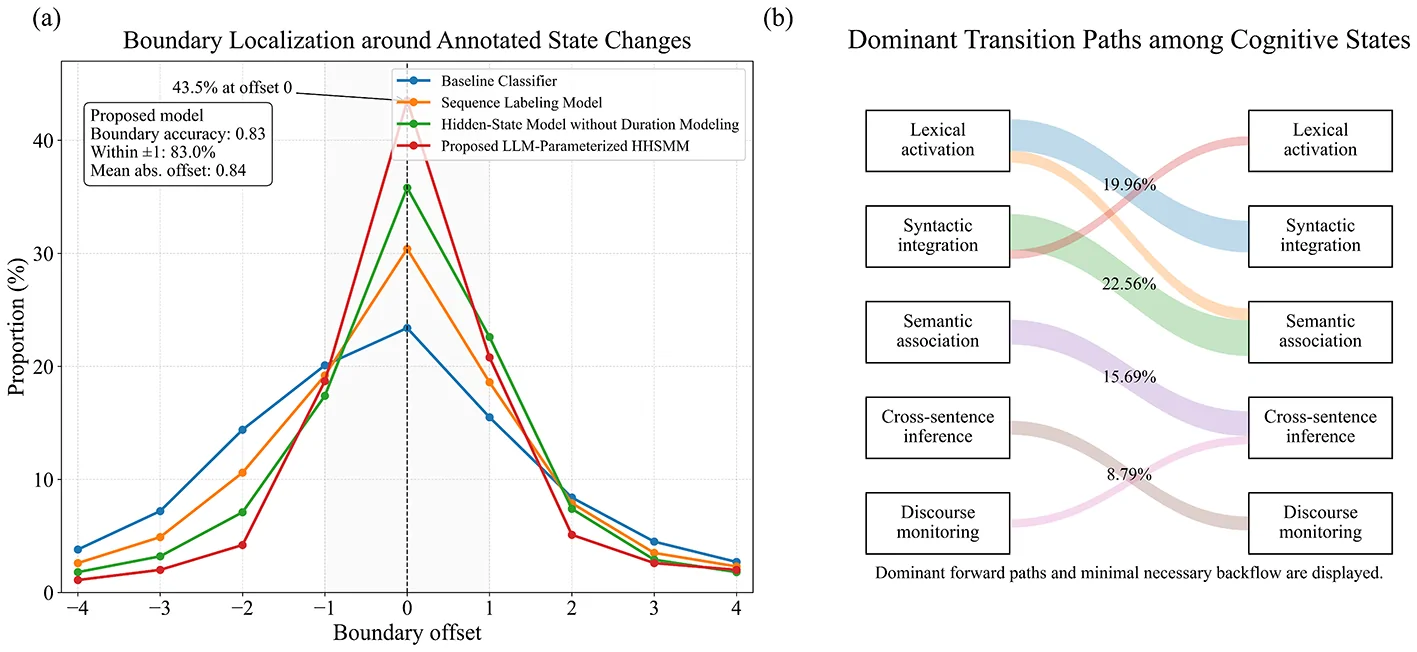
Cognitive-state boundary localization and stage-switching structure. **(a)** distribution of boundary-positioning deviation. **(b)** dominant phase-switching paths.

In Figure 5, the proposed model predicts the exact boundary position for 43.5% of cases, compared with 35.8% for the no-duration hidden-state model, 30.4% for the sequence-labeling model, and 23.4% for the baseline classifier. Predictions within +/-1 reading unit reach 83.0%, consistent with the reported boundary accuracy of 0.83. The mean absolute offset decreases to 0.84, lower than 1.02, 1.20, and 1.42 for the comparison models. These results show that explicit duration modeling and discourse-structure constraints reduce boundary drift caused by local fluctuations. The dominant forward paths are Lexical Activation to Syntactic Integration (19.96%), Syntactic Integration to Semantic Connection (22.56%), Semantic Connection to Cross-sentence Inference (15.69%), and Cross-sentence Inference to Discourse Monitoring (8.79%). Reverse paths are less frequent, which is consistent with the gradual movement from local access toward discourse-level monitoring in continuous reading. In Figure 5b, each percentage uses the total number of decoded state switches on the test set as its denominator. Switches are counted between adjacent decoded segments rather than between adjacent positions, and self-transitions within a segment are excluded because persistence is modeled through the duration distribution. Table 3 reports the complete five-by-five transition matrix. Forward transitions to the next state in the processing order account for 67.0 percent of all switches, reverse transitions account for 18.9 percent, and transitions that skip one or more states account for 14.1 percent, so the lower frequency of reverse paths is a quantitative result rather than a descriptive impression. Under the relaxed setting with a uniform transition prior and without structural constraints, the forward share remains at 58.9 percent, as reported in Table 4, which shows that forward dominance is retained when these assumptions are relaxed. A paired text-level bootstrap over 1,000 resamples places the 95 percent confidence interval of the boundary-accuracy gap between the proposed model and the no-duration hidden-state model between 0.041 and 0.057, which supports attributing the boundary-localization gains to duration modeling and discourse-structure constraints.

**Table 3:** Complete transition matrix of decoded states on the test set.

From\To	LA	SI	SC	CI	DM
LA	—	19.96	4.6	1.2	0.4
SI	4.1	—	22.56	4.5	1
SC	1.9	5.2	—	15.69	2.4
CI	0.4	1.1	3.8	—	8.79
DM	0.1	0.2	0.5	1.6	—

LA, lexical activation; SI, syntactic integration; SC, semantic connection; CI, cross-sentence inference; DM, discourse monitoring.

**Table 4:** Sensitivity analysis results under relaxed model assumptions.

Setting	Macro-F1	Boundary Acc	Fwd Trans (%)
Full model	0.860	0.830	67.0
w/o duration term	0.822	0.781	63.4
w/o discourse coherence constraint	0.831	0.762	58.9
Random order initialization	0.854	0.821	61.2
Uniform prior re-estimation	0.857	0.826	64.8

Boundary Acc, boundary accuracy; Fwd Trans, forward transition ratio.

Table 4 reports the sensitivity results. Removing the duration term lowers macro-F1 to 0.822 and boundary accuracy to 0.781, while the dominant forward-transition share remains at 63.4 percent. Removing the discourse-structure consistency constraint lowers boundary accuracy to 0.762 with a forward share of 58.9 percent. Randomizing the ordering-based initialization before training leaves macro-F1 at 0.854, and refitting the transition matrix from a uniform prior leaves macro-F1 at 0.857. Under every relaxed setting, the progression from lexical activation toward discourse monitoring remains the majority pattern in the decoded transitions, which demonstrates that the progression is estimated from the annotated data and is not a mechanical consequence of the imposed transition, duration, or consistency constraints.

### 4.3 State duration modeling

Duration analysis focuses on the five cognitive states and compares the proposed model with the three baselines in terms of mean absolute error. State persistence was examined through the number of decoded segments, mean duration, and the proportion of long-span segments. For the MAE calculation, each decoded segment is matched to the annotated segment with which it shares the largest positional overlap within the same passage, and the absolute difference between their durations in reading units is recorded. The state-level MAE averages these differences over all matched segments of the state across all test passages, and the overall MAE is the unweighted mean of the five state-level MAEs. Table 5 summarizes these results.

**Table 5:** Duration characteristics and fitting errors for different cognitive states.

State	Segments	Mean Dur.	Long-span ratio ≥ 4	BC MAE	SLM MAE	HS MAE	Proposed MAE
Lexical activation	8,904	1.74	8.60%	1.08	0.95	0.89	0.66
Syntactic integration	8,400	2.31	18.90%	0.96	0.82	0.77	0.56
Semantic connection	6,142	2.87	29.70%	0.89	0.74	0.69	0.51
Cross-sentence inference	3,545	3.96	51.80%	0.81	0.66	0.58	0.46
Discourse monitoring	2,306	4.62	63.40%	0.74	0.58	0.47	0.41
Overall	29,297	2.64	25.50%	0.9	0.75	0.68	0.52

Dur. denotes duration in reading units. Long-span ratio refers to the proportion of state segments with duration greater than or equal to four reading units. BC denotes Baseline Classifier. SLM denotes Sequence Labeling Model. HS denotes Hidden-State Model without Duration Modeling.

Table 5 indicates a clear temporal gradient. Lexical activation has the shortest mean duration (1.74) and the lowest long-span ratio (8.6%), which is consistent with its role in local word access. Syntactic integration and semantic connection last longer, with mean durations of 2.31 and 2.87 and long-span ratios of 18.9% and 29.7%. Cross-sentence inference and discourse monitoring show stronger persistence, with mean durations of 3.96 and 4.62 and long-span ratios of 51.8% and 63.4%. The proposed model yields MAEs of 0.66, 0.56, 0.51, 0.46, and 0.41 across the five states and an overall MAE of 0.52, lower than the no-duration hidden-state model (0.68), the sequence-labeling model (0.75), and the baseline classifier (0.90). Because the annotated durations are produced under guidelines that encode sequence continuity and longer spans for higher-level states, this improvement reflects a close fit to the annotation scheme rather than direct validation of an underlying cognitive mechanism, which awaits behavioral and neurocognitive evidence.

### 4.4 Cross-sentence reasoning and discourse monitoring

The next analysis examines the two states most closely associated with deep discourse comprehension: cross-sentence inference and discourse monitoring. The analysis considers changes in discourse complexity and context-span expansion under the same test set and evaluation criteria. The Low, Medium, and High groups in Figure 6a are the discourse-complexity groups defined in **Section 3.1**, with 47, 81, and 52 test passages respectively. The context spans in Figure 6b are defined operationally: the Local span is the current sentence alone, the Adjacent span adds one preceding sentence, the Short-range span adds two preceding sentences, and the Long-range span adds four preceding sentences. Performance gain is the F1 difference between the proposed model and the no-duration hidden-state model computed on the same passages under the same span. A paired text-level bootstrap over 1,000 resamples places the 95 percent confidence interval of the Long-range gain between 0.041 and 0.065 for cross-sentence inference and between 0.046 and 0.072 for discourse monitoring, and the gain increase from the Local to the Long-range span remains positive in 96.4 percent of resamples for both states. Figure 6 reports the results.

**Figure 6:**
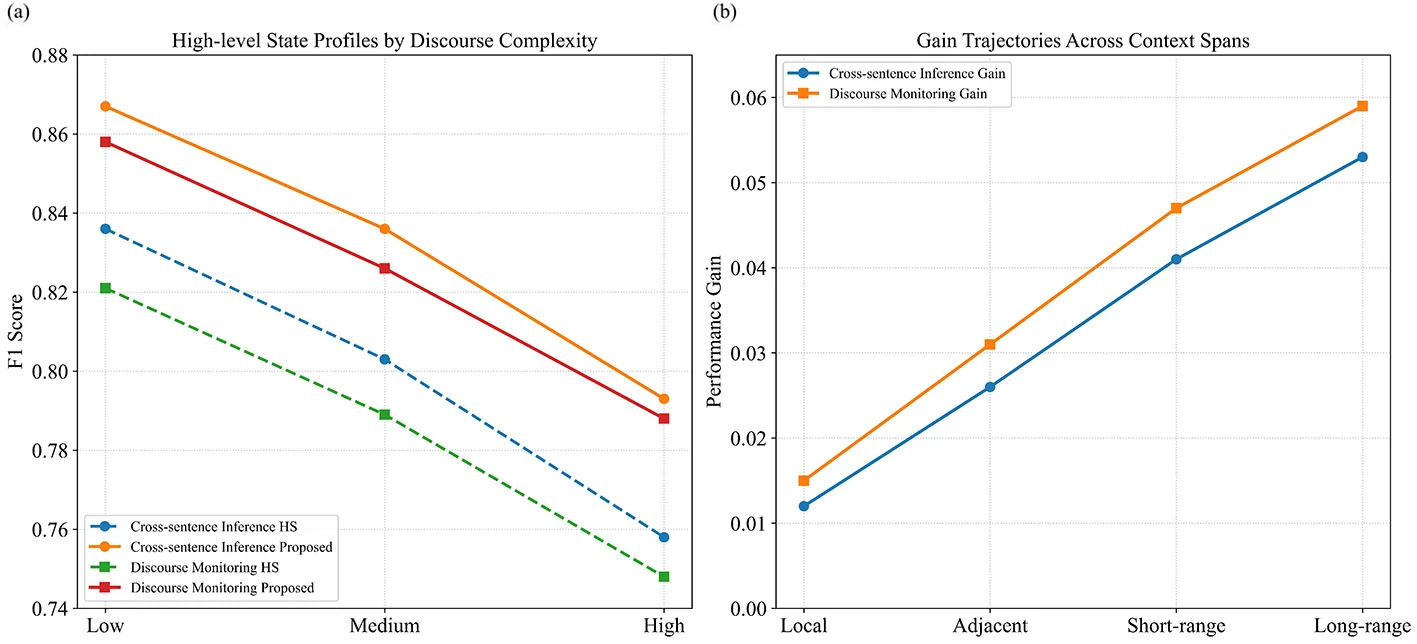
Deep-comprehension characteristics of higher-level cognitive states. **(a)** higher-level state recognition under different discourse complexities. **(b)** higher-level state gains under different context spans.

Figure 6 shows that both high-level states become harder to identify as discourse complexity increases. Cross-sentence inference in the no-duration hidden-state model decreases from 0.836 to 0.758, while the proposed model decreases from 0.867 to 0.793. Discourse monitoring in the no-duration model decreases from 0.821 to 0.748, whereas the proposed model decreases from 0.858 to 0.788. Although complexity still reduces performance, the proposed model remains stronger at every level. Context expansion produces the opposite pattern: the gain for cross-sentence inference rises from 0.012 to 0.053, and the gain for discourse monitoring rises from 0.015 to 0.059. This result is consistent with model-brain studies showing that language-model representations become more informative when they capture discourse-level context rather than isolated words or sentences (Goldstein et al., 2022; Schrimpf et al., 2021; Yu et al., 2024). These results show that broader context improves the prediction of annotated higher-level state labels. They are consistent with the view that human high-level reading states depend on accumulated context and global coherence, but they do not establish that dependence, and the connection to model-brain studies remains indirect because the present study contains no neural data or model-brain alignment analysis.

### 4.5 Interpretability of cognitive trajectories

To examine interpretability, discourse position, dominant cognitive state, and hierarchical support were analyzed together. The goal was to determine whether the decoded trajectory forms an internally coherent process representation rather than a sequence of isolated labels. Figure 7 presents the explanatory trajectory.

**Figure 7:**
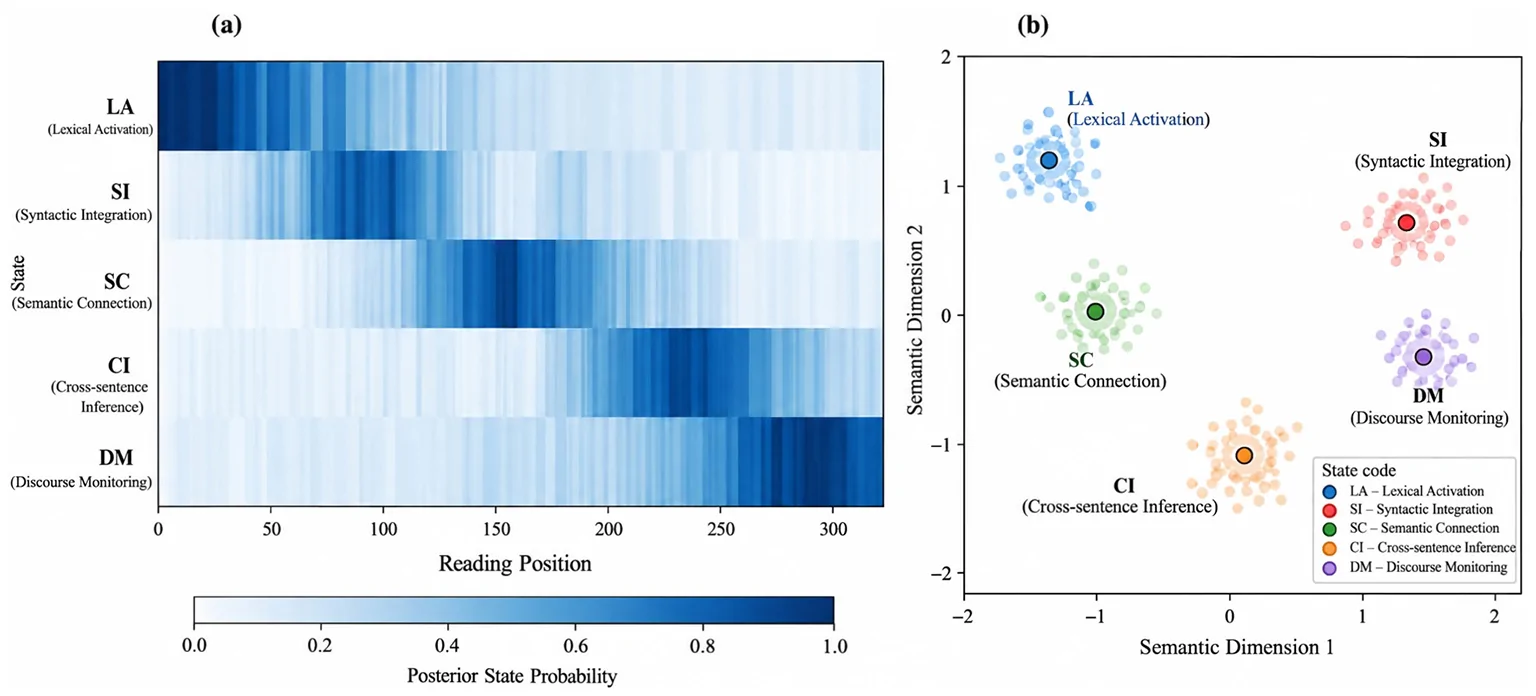
Cognitive state sequence and semantic space distribution of the five cognitive states. **(a)** Cognitive state sequence across reading positions. **(b)** Cognitive states in semantic space.

The left panel of Figure 7 combines a discourse-stage band, a dominant-state band, and three hierarchical support curves. Reading positions 0-16 are dominated by lexical activation and syntactic integration; lexical support decreases from 78 to 42, whereas sentence support increases from 17 to 48. Positions 41-67 shift toward cross-sentence inference, with discourse-level support increasing from 43 to 53. Positions 67-100 are concentrated in discourse monitoring, where discourse-level support remains between 66 and 78 and dominant-state strength remains between 0.81 and 0.85. The right panel further shows systematic differentiation across states. Lexical activation is located early in the passage, with an average position of 12.4, hierarchical center of 1.33, mean duration of 1.74, and F1 of 0.901. Discourse monitoring appears later, with an average position of 86.1, hierarchical center of 2.68, mean duration of 4.62, and F1 of 0.824. The joint shift in position, hierarchy, and duration indicates that the model has learned an interpretable division of labor from local recognition to discourse-level regulation.

### 4.6 Causal, cross-architecture, and pooling analyses

#### 4.6.1 Causal parameter generation

To align the parameter generator with the incremental left-to-right nature of human reading, we built a causal variant in which the encoder applies a causal attention mask and the context window is restricted to the current sentence and the four preceding sentences. On the test set, the causal variant reaches a macro-F1 of 0.838 against 0.860 for the bidirectional model, a boundary accuracy of 0.802 against 0.830, and a duration MAE of 0.58 against 0.52. The degradation concentrates in the higher-level states: cross-sentence inference falls from 0.832 to 0.791 and discourse monitoring falls from 0.824 to 0.783, whereas lexical activation changes only from 0.901 to 0.894 and syntactic integration moves from 0.882 to 0.866. Mean boundary detection latency, measured as the delay in reading positions between the annotated boundary and the first decoded position of the new state, rises from 0.9 to 1.6. This pattern quantifies the cost of removing future context: high-level states rely more on upcoming disambiguating information, while low-level states remain stable under strictly incremental encoding, which matches the temporal profile of online reading.

#### 4.6.2 Alternative LLM architectures

To test whether the parameter-generation mechanism is model-agnostic, we replaced the encoder with representatives of three further paradigms: the bidirectional BERT-base, native causal decoders from the Qwen 3 and Gemma 4 families at two sizes each, and the state-space Mamba 3 at two sizes.

Table 6 reports the results on the test set, with the context window fixed at two preceding and two following sentences for the bidirectional models and at four preceding sentences for the causal and state-space models. All architectures reach macro-F1 between 0.818 and 0.867, and the ranking tracks representational capacity rather than architectural family: the two four-billion-parameter decoders reach 0.867 and 0.864, DeBERTa-v3-base reaches 0.860, and Mamba 3 at 1.3 billion parameters reaches 0.845. Widening the context window of DeBERTa-v3 from one to two surrounding sentences raises macro-F1 from 0.847 to 0.860, while a further widening to four surrounding sentences yields 0.862, and the causal decoders show the same saturation pattern. These results show that the gains reported in this paper stem from the hybrid parameter-generation architecture rather than from a specific encoder, and that moderate context windows capture most of the usable discourse signal.

**Table 6:** Encoder architecture performance comparison.

Encoder	Architecture paradigm	macro-F1	Boundary accuracy	Duration MAE
BERT-base	Bidirectional encoder	0.831	0.794	0.61
DeBERTa-v3-base	Bidirectional encoder	0.860	0.830	0.52
Qwen3-0.6B	Causal decoder	0.842	0.809	0.56
Qwen3-4B	Causal decoder	0.867	0.835	0.50
Gemma4-1B	Causal decoder	0.839	0.806	0.57
Gemma4-4B	Causal decoder	0.864	0.833	0.51
Mamba3-130M	State-space model	0.818	0.783	0.63
Mamba3-1.3B	State-space model	0.845	0.813	0.55

#### 4.6.3 Subword-to-word pooling strategies

To assess how subword aggregation affects state modeling, we replaced mean-pooling with four alternatives: attention-weighted pooling with a learned query per state, layer-wise scalar mixing across the encoder layers, element-wise max-pooling, and first-and-last subword concatenation followed by a linear projection. Layer-wise scalar mixing performs best, raising macro-F1 from 0.860 to 0.871 and boundary accuracy from 0.830 to 0.845, and lowering duration MAE from 0.52 to 0.48. Attention-weighted pooling reaches a macro-F1 of 0.866 and first-and-last concatenation reaches 0.863, and both outperform mean-pooling on every metric, whereas max-pooling falls below mean-pooling on boundary accuracy at 0.818. The pattern indicates that naive averaging dilutes the morphological and syntactic signals carried by individual subwords and by lower encoder layers, and that preserving layer-specific and position-specific subword information sharpens state boundaries and duration estimates. The main-text results are retained with mean-pooling for comparability with the original protocol, and the scalar-mixing configuration is reported as the recommended setting.

In Figure 7, the lexical, sentential, and discourse support shares are the normalized layer contributions defined for Figure 4b, averaged within one hundred equal discourse-position bins across the test set. The hierarchical center of a state is the mean layer index of its dominant support, with the lexical, sentence, and discourse layers indexed as one, two, and three. Discourse stages are contiguous ranges of the normalized position axis delimited by changes in the modal dominant state, and the state band in Figure 7a reports the modal dominant state within each bin across all test passages rather than a single passage or an illustrative trajectory. Because the support curves, the state band, and the duration values all derive from the same annotations and decoded sequences, their mutual agreement reflects the internal coherence of the model outputs rather than independent cognitive validation. The category-specific F1 values in Figure 7b are included to show where recognition errors concentrate along the trajectory, and they describe recognition performance rather than an intrinsic property of the states. Across the test set, the discourse-comprehension consistency correlation was 0.76.

## 5 Discussion

The findings indicate that large-model contextual representations become more useful for reading-process modeling when they are constrained by a cognitive state architecture. The LLM encoder supplies rich semantic and discourse information, but the hierarchical semi-Markov layer gives this information temporal form. This combination is important for the Research Topic because it treats a large model not as a static text classifier but as a computational platform for modeling adaptive cognition.

The results also clarify why duration matters. Lexical activation is brief and locally triggered, whereas cross-sentence inference and discourse monitoring persist across longer spans. This pattern resembles the temporal organization emphasized in predictive-processing and efficient-coding accounts, where higher-level representations integrate information over broader contexts. The proposed model operationalizes this idea through explicit state-duration parameters rather than through *post hoc* interpretation.

At the same time, the model should not be overread as a direct neural model. It does not claim biological equivalence, nor does it include neuronal plasticity or embodied sensorimotor interaction. Its contribution is narrower: it provides a bridge between discourse-comprehension theory, probabilistic state modeling, and large-model contextual encoding. This positioning is consistent with the special issue's interest in using large models to generate and test hypotheses about adaptive cognition.

Several limitations remain. The corpus is based mainly on standardized English reading materials, and the state labels depend on expert annotation. The main experiments rely on a bidirectional encoder with a symmetric context window, and the causal analysis in **Section 4.6** shows that strictly incremental encoding lowers the recognition of higher-level states, so online deployment of the framework requires the causal configuration. Direct behavioral and neurocognitive evidence is needed to validate the decoded trajectories against actual reading processes.

## 6 Conclusion

This study proposed a brain-inspired, LLM-parameterized hierarchical hidden semi-Markov model for modeling cognitive states in complex English discourse reading. The proposed model unifies state category, boundary, persistence, and cross-level coupling within a single framework. The experimental results confirm stable improvements across all four evaluation tasks. The results suggest that large language models can be used as adaptive parameter generators for cognitive-process modeling, provided that their representations are coupled with interpretable temporal structure. The study is still limited by its reliance on standardized reading materials and annotation-based state labels. Future work will incorporate eye-tracking and neural evidence, test online and reader-specific adaptation, and explore whether the model can serve as a cognitive-core module for embodied language-learning agents in more open environments.

## Data Availability

The original contributions presented in the study are included in the article/supplementary material, further inquiries can be directed to the corresponding author.
